# Genetic evidence for causal effects of leukocyte counts on risk for rheumatoid arthritis

**DOI:** 10.1038/s41598-023-46888-1

**Published:** 2023-11-26

**Authors:** Jin-Mei You, Yao-Chen Zhang, Ke-Yi Fan, Shang-Kai Bai, Zi-Yu Zhang, He-Yi Zhang, Ting Cheng, Yue-Hong Huo, Cai-Hong Wang, Xiao-Feng Li, Sheng-Xiao Zhang

**Affiliations:** 1 Department of Clinicallaboratory, The Second Hospital of Shanxi Medical University, Taiyuan, Shanxi Province China; 2 Shanxi Provincial Key Laboratory of Rheumatism Immune Microecology, Taiyuan, Shanxi Province China; 3 Key Laboratory of Cellular Physiology at Shanxi Medical University, Ministry of Education, Taiyuan, Shanxi Province China; 4 Department of Rheumatology, The Second Hospital of Shanxi Medical University, Taiyuan, 030001 Shanxi Province China; 5 Department of Rheumatology, The Fifth People’s Hospital of Datong, Datong, Shanxi Province China

**Keywords:** Infection, Lymphocytes, Epigenomics, Osteoimmunology

## Abstract

Rheumatoid arthritis (RA) is an autoimmune disease characterized by the accumulation of leukocytes and inflammatory mediators within the synovial tissue. Leukocyte counts are proposed to play a role in the pathogenesis of RA. However, the causality remains unclear. To investigate the causal relationship between various leukocytes and RA by implementing two-sample univariable Mendelian Randomization (MR) and multivariable MR. MR analysis was performed using respective genome-wide association study (GWAS) summary statistics for the exposure traits (eosinophil counts, neutrophil counts, lymphocyte counts, monocyte counts, basophil counts, and white blood cell counts) and outcome trait (RA). Summary statistics for leukocytes were extracted from the Blood Cell Consortium meta-analysis and INTERVAL studies. Public GWAS information for RA included 14,361 cases and 43,923 controls. Inverse variance weighted, weighted median, MR-Egger regression, MR pleiotropy residual sum and outlier, and multivariable MR analyses were performed in MR analysis. Univariable MR found elevated eosinophil counts (OR 1.580, 95% CI 1.389–2.681, *p* = 1.30 × 10^–7^) significantly increased the risk of RA. Multivariable MR further confirmed that eosinophil counts were a risk factor for RA. Increased eosinophils were associated with higher risk of RA. Further elucidations of the causality and mechanisms underlying are likely to identify feasible interventions to promote RA prevention.

## Introduction

Rheumatoid arthritis (RA) is one of the most common chronic autoimmune diseases that affect many joints and causes cartilage injury, bone damage, and systemic diseases with high mortality and morbidity^1^. The prevalence of RA increases with age, and major societal evidence demonstrates steady growth in the incidence of RA in recent decades^2^. The consensus of RA is that individuals lose self-tolerance and begin to produce autoantibodies in genetically predisposed individuals at the early stage. Eventually, the disease transformed from asymptomatic autoimmunity to synovial inflammation. The tissue responds with a maladaptive wound-healing response, leading to irreversible tissue injury targeting tendons, cartilage, and bone^3^. The genetic factors and multiple environmental include lifestyle, behavioral risk factors, and microbiome, are considered risk factors for disease^4^. And genetic susceptibility is apparent in RA, and almost 50% of RA risk is attributable to hereditary factors^5^.

Leukocytes, derived from hematopoietic stem cells, is comprised of five subtypes: lymphocyte, basophil, eosinophil, neutrophil, and monocyte. Several studies have evaluated associations between leukocytes and RA^6,7^. Eosinophilia is a predictor of poor clinical outcomes in early arthritis. For example, a prospective cohort study indicates that patients with eosinophilia show a higher disease activity^8^. Patients with RA are more likely to have eosinophilia^9,10^, and eosinophil-lymphocyte ratio levels were increased in RA patients^6^. While current evidence from observational studies is not entirely consistent on the relation between leukocytes and RA risk. A prospective observational study reported no significant difference in clinical features between RA with persistent eosinophilia and those without eosinophilia^11^. Besides, emerging evidence indicates the protective effect that the eosinophil subset can have on RA^12^. Nevertheless, cross-sectional studies do not support a causal association. Besides, observational studies are susceptible to biases such as potential confounding, measurement error, and reverse causation^13^. The above indicates that new methods are urgently needed to elucidate the relationship between various leukocytes and RA.

Mendelian randomization (MR) is a novel method to research the association between exposures and outcomes using instrumental variables (IVs) to determine whether there is an association and causal role. The MR method is less likely to be disturbed by potential confounding factors because the parental allele is randomly distributed to the offspring^14^. Because single-nucleotide polymorphism (SNP) alleles were distributed before the onset of meiosis, the risk of reverse causality will be minimized^15^. Besides, to research the direct effects of RA and various leukocytes, we conducted multivariable MR (MVMR) because the different leukocytes are not independent. MVMR can estimate the direct effect instead of the total effect that each exposure plays a role in the outcome^16^.

In our study, we conducted a comprehensive two-sample MR analysis and MVMR analysis to evaluate the causality of white blood cell counts in the risk of RA.

## Methods

### Summarized statistics of leukocyte traits

GWAS data of leukocyte traits was derived from the Blood Cell Consortium meta-analysis^17^, which includes data from European ancestry individuals (http://www.mhi-humangenetics.org/en/resources/). Age, sex, age-squared and cohort-specific covariates were corrected in the GWAS study. Six phenotypes (eosinophils, neutrophils, lymphocytes, monocytes, basophils, and white blood cell counts) were selected as exposure in this study. GWAS summary data for the leukocyte traits used for external validation was obtained from the European Bioinformatics Institute (EBI) database^18^. The large GWAS was performed in the UK Biobank and INTERVAL studies, including 563,946 European-ancestry participants. 29.5 million genetic variants for association with 36 red cell, white cell, and platelet properties were tested.

### Summarized statistics of RA

The GWAS data of RA were retrieved from a public GWAS website (https://gwas.mrcieu.ac.uk/, ID: ebi-a-GCST90013534). The GWAS information from the European people involved in 14,361 cases and 43,923 controls and 13,108,512 SNPs^19^.

### Selection of IVs

**Figure 1:**
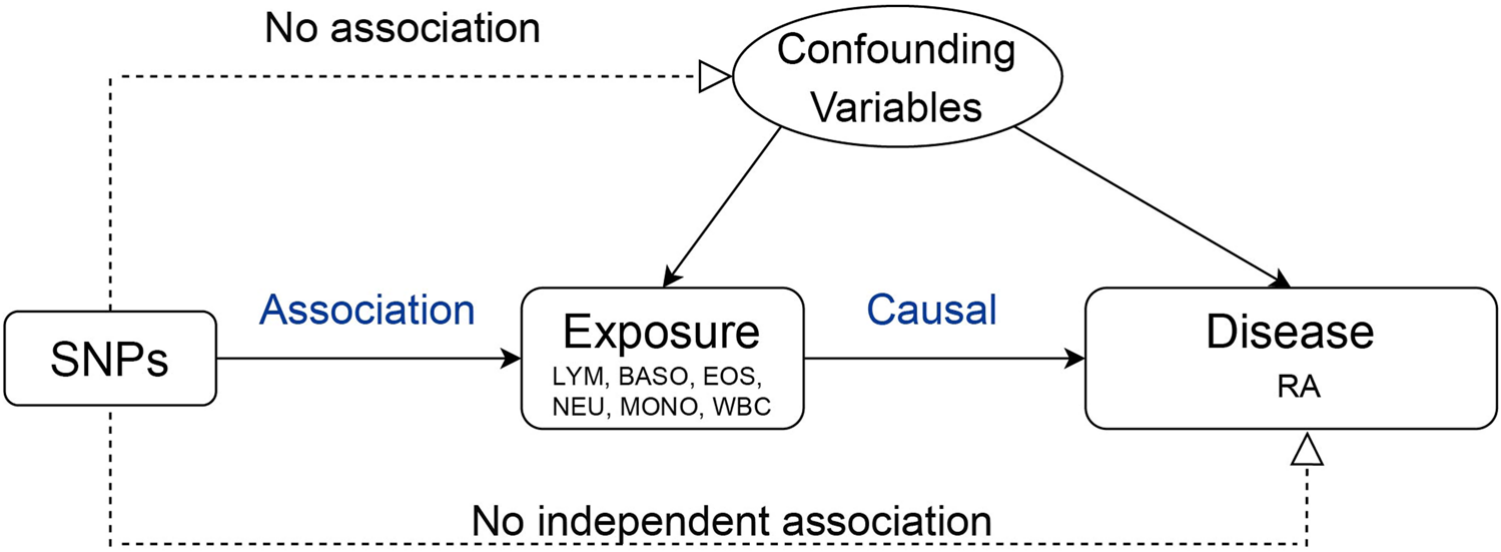
Directed acyclic graph (causal diagram) of Mendelian Randomization. LYM, lymphocyte counts; BASO, basophil counts; EOS, eosinophil counts; NEU, neutrophil counts; MONO, monocyte counts; WBC, white blood cell counts; RA, rheumatoid arthritis.

IVs extracted meet three criteria to ensure the credibility of this study: SNPs are significantly associated with leukocytes; SNPs have to be independent of confounders affecting leukocytes and the RA; SNPs have to influence the RA through leukocytes rather than through other ways^20^ (Fig. 1).

First, genome-wide significant (*p* < 5 × 10^–8^) SNPs associated with leukocytes were extracted as the instrumental variables. These SNPs were independent by excluding SNPs in linkage disequilibrium (r^2^ < 0.01, clumping window = 10,000 kb). Besides, we queried and removed SNPs associated with confounders in PhenoScanner to exclude potential pleiotropic effects. We also considered and removed palindromic SNPs. Finally, the F statistic was calculated to judge the strength of the genetic instrument variables in the results. An F-statistic ≥ 10 indicates strong relation between the instrumental variables and exposure^21^.

### MR analysis

To assure the consistency of data, we harmonized the statistics of leukocytes and RA and subsequently conducted them. Subsequently, we conducted various MR methods to evaluate the causal associations between leukocytes and RA. For the univariate MR analysis, inverse-variance weighted (IVW) was the primary method. The MR-Egger regression and weighted median were also used to evaluate causal relationships. The IVW acquires a robust causal estimate, but it is biased when the SNPs are pleiotropy because of the ignorance of intercept^22^. And the other methods were used for further supplementary analyses. The MR-Egger regression provides estimates after correcting for pleiotropy, although the MR-Egger method is weak in statistical ability^23^. The weighted median also attains robust causal estimates if half of the instrument variables analyzed are invalid^24^.

Besides, we conducted MVMR analysis to research the direct effects of various white blood cell counts (lymphocyte, neutrophil, monocyte, eosinophil, and basophil) on RA. MVMR analysis is an extension of MR analysis that probes the causal effects of multiple factors. It is effective in cases where two or more exposures are related and can help identify whether some directions have a causal impact on the outcome or whether one exposure may be mediated through the effect of the others^25^.

### Sensitivity analysis

We conducted various approaches to detect the presence of heterogeneity or pleiotropy. In the study, the *p*-value of Cochrane’s Q was used to assess the degree of heterogeneity, where *p* > 0.05 suggested no heterogeneity. MR-Egger regression intercept was used to determine the horizontal pleiotropy. The MR pleiotropy residual sum and outlier (MR-PRESSO) method was applied to detect outlier SNPs in IVW linear regression and correct the MR estimation by removing these outliers. Moreover, a leave-one-out analysis was conducted to avoid a single SNP causing horizontal pleiotropy. Finally, funnel, scatter, and leave-one-out plots were also constructed for visual inspection.

### Statistical analysis

Bonferroni correction was used for multiple-comparison correction. MR results with *p* < 8.33 × 10^–3^ (0.05/6) and *p* < 0.05 were considered to have statistically significant for univariable MR and multivariable MR, respectively. The estimated relative risk was the odds ratio and 95% confidence interval (CI). All statistical analyses mentioned above were conducted using R (version 4.2.2), and the “TwoSampleMR” package, the “MVMR” package, and the “MRPROSSO” package were used for MR analysis.

### Ethics approval and consent to participate

The summary statistics don’t contain any personal information and the original GWAS data of leukocyte counts and RA have obtained ethical approval from relevant ethics review committees.

## Results

### The character of SNP and participants for analysis

**Figure 2:**
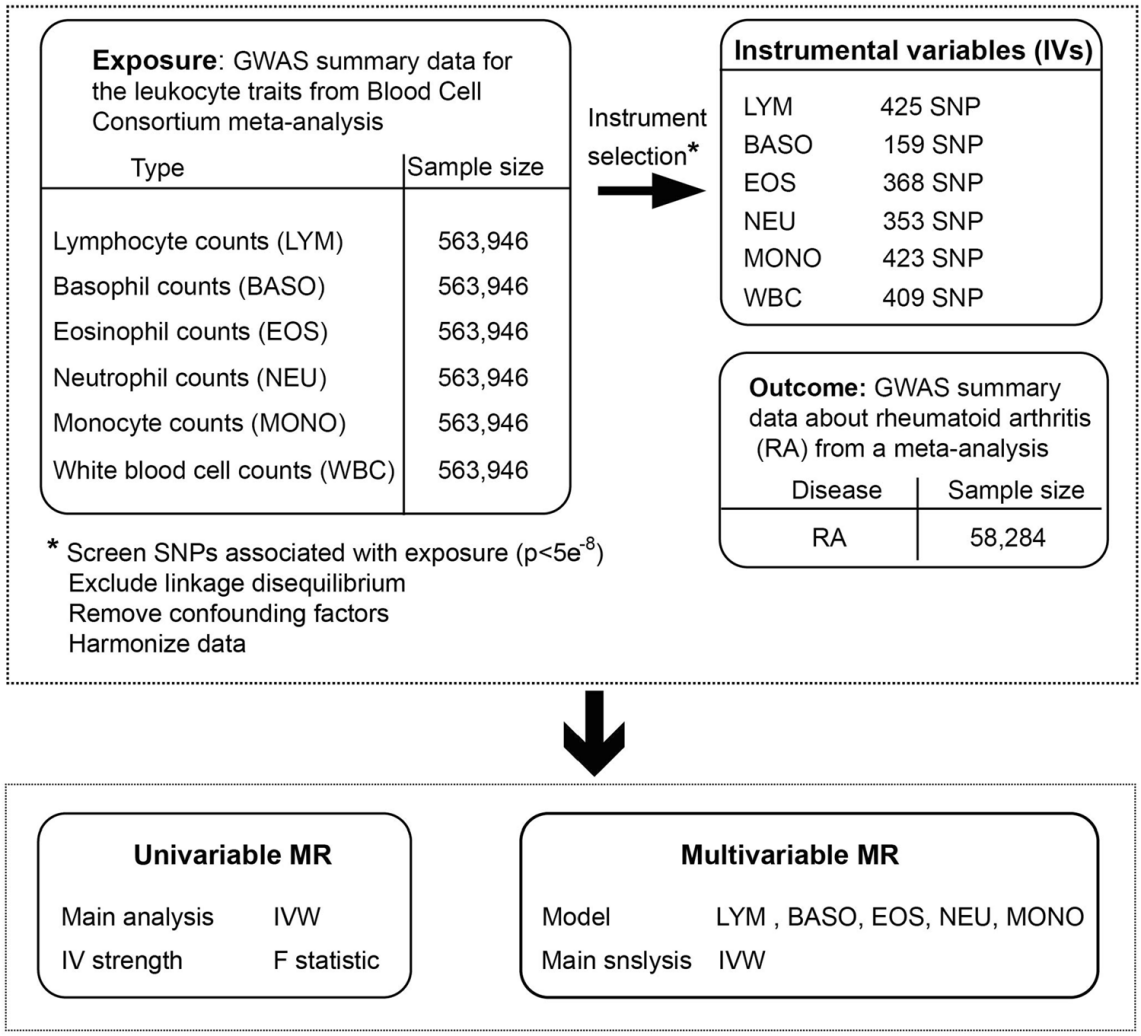
Flow chart of the study design. IV, instrumental variables; IVW, inverse-variance weighted.

We included genome-wide significant SNPs (*p* < 5 × 10^–8^). Then, these SNPs were clustered based on linkage disequilibrium (r^2^ < 0.01). The mean F-statistic ranged from 119.57 to 176.34, indicating a low risk of weak instrument bias. At the same time, the harmonized algorithm removes all SNPs that have palindromic structures. In the SNP associated with the leukocytes counts after the screening, 425, 353, 423, 368, 159, and 409 SNPs were included as candidate IVs for MR analysis of total lymphocyte, neutrophil, monocyte, eosinophil, basophil counts and white blood cell counts with RA, respectively (Fig. 2) (Tables S1–S6)^26^.

### The causal effect of leukocytes on RA

**Figure 3:**
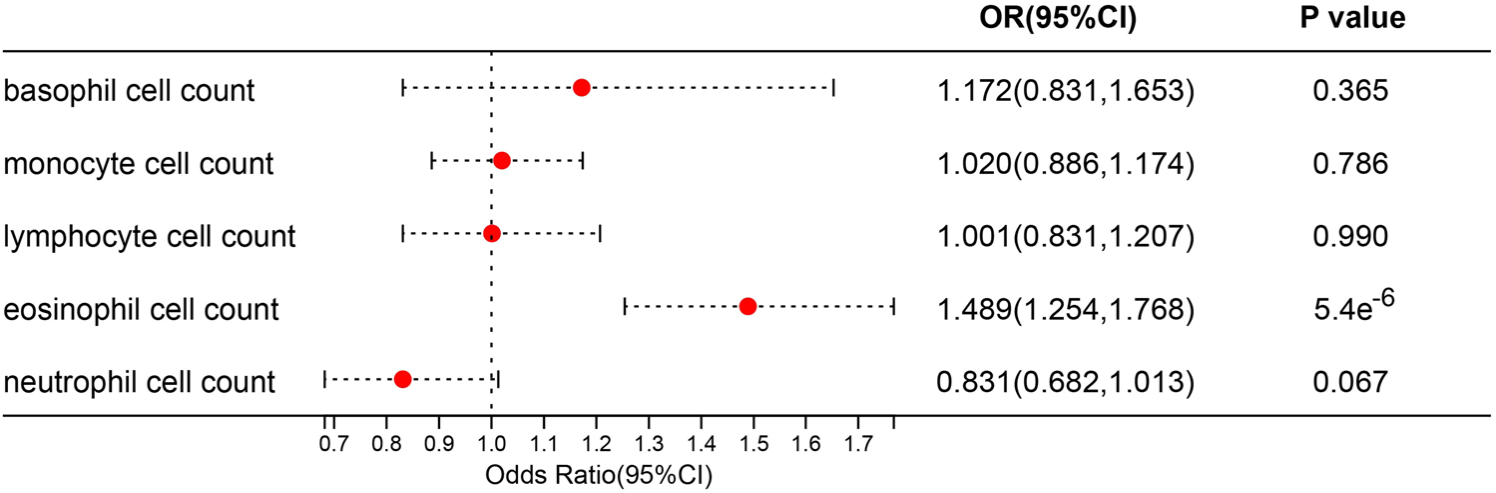
Multivariable MR analysis forest plot: effect of multiple risk factors on RA. OR, odds ratio; CI, confidence interval.

IVW, MR-Egger, and weighted median regression were used to estimate the causal relationship between genetically predicted RA and leukocytes counts. In univariate analysis, eosinophil counts (OR 1.580, 95% CI 1.389–2.681, *p* = 1.30 × 10^–7^) are risk factors for RA. There was no significant association between basophil counts (OR 1.195, 95% CI 0.964–1.480, *p* = 0.103), neutrophil counts (OR 1.199, 95% CI 0.942–1.526, *p* = 0.140), lymphocyte counts (OR 0.897, 95% CI 0.773–1.040, *p* = 0.149), monocyte counts (OR 1.068, 95% CI 0.981–1.162, *p* = 0.817), and white blood cell counts (OR 1.149, 95% CI 0.981–1.345, *p* = 0.085) and RA (Table 1). In multivariate analysis, only a causal relationship exists between eosinophil counts and RA (*p* = 5.40 × 10^–6^) (Fig. 3). To sum up, we conclude that eosinophil counts are the major risk factor for RA.

**Table 1:** Univariable MR estimates of types of blood leukocyte counts on the risk of RA.

Exposure	MR methodology	N of SNP	Effect estimates			Test of heterogeneity		Test of pleiotropy	
			OR	95% CI	*P* value	Cochrane *Q* test	P_heterogeneity_	MR-Egger intercept	P_pleiotropy_
Monocyte counts	IVW	423	1.068	0.981–1.162	0.817	958.741	9.50e^−44^		
	MR Egger	423	1.018	0.875–1.185	0.966	957.498	8.91e^−44^	0.001591	0.460
	Weighted median	423	1.002	0.901–1.115	0.128				
basophil counts	IVW	159	1.195	0.964–1.480	0.103	592.003	1.94e^−51^		
	MR Egger	159	1.293	0.837–1.998	0.248	591.365	1.26e^−51^	−0.002079	0.681
	Weighted median	159	1.372	1.118–1.684	0.002				
Eosinophil counts	IVW	368	1.580	1.389–2.681	1.300E−07	2720.126	0		
	MR Egger	368	1.930	1.040–1.350	0.000	2705.864	0	−0.006277	0.166
	Weighted median	368	1.185	1.333–1.873	0.011				
Neutrophil counts	IVW	353	1.199	0.942–1.526	0.140	4206.04	0		
	MR Egger	353	1.424	0.875–2.318	0.155	4198.423	0	−0.004616	0.425
	Weighted median	353	0.859	0.746–0.988	0.033				
Lymphocyte counts	IVW	425	0.897	0.773–1.040	0.149	2407.283	9.69e^−274^		
	MR Egger	425	0.975	0.717–1.325	0.871	2405.165	9.66e^−274^	−0.002322	0.542
	Weighted median	425	0.986	0.863–1.127	0.841				
White blood cell counts	IVW	409	1.149	0.981–1.345	0.085	2408.376	4.74e^−280^		
	MR Egger	409	1.327	0.962–1.832	0.086	2402.382	2.35e^−279^	−0.003875	0.314
	Weighted median	409	1.144	0.992–1.319	0.063				

*OR* odds ratio, *95% CI* 95% confidence interval.

### The causal effect of eosinophil counts on RA in validation stage

In the validation stage, we successfully replicated the MR results of eosinophil counts on RA. Univariable MR results demonstrated that eosinophil counts had a significant causal effect on RA (OR 1.206, 95% CI 1.040–1.398, *p* = 0.013). The scatter plot and plot of leave-one-out analyses were displayed in Fig. S1. The information of IVs of eosinophil counts is shown in Table S7.

### Sensitivity analysis validation

**Figure 4:**
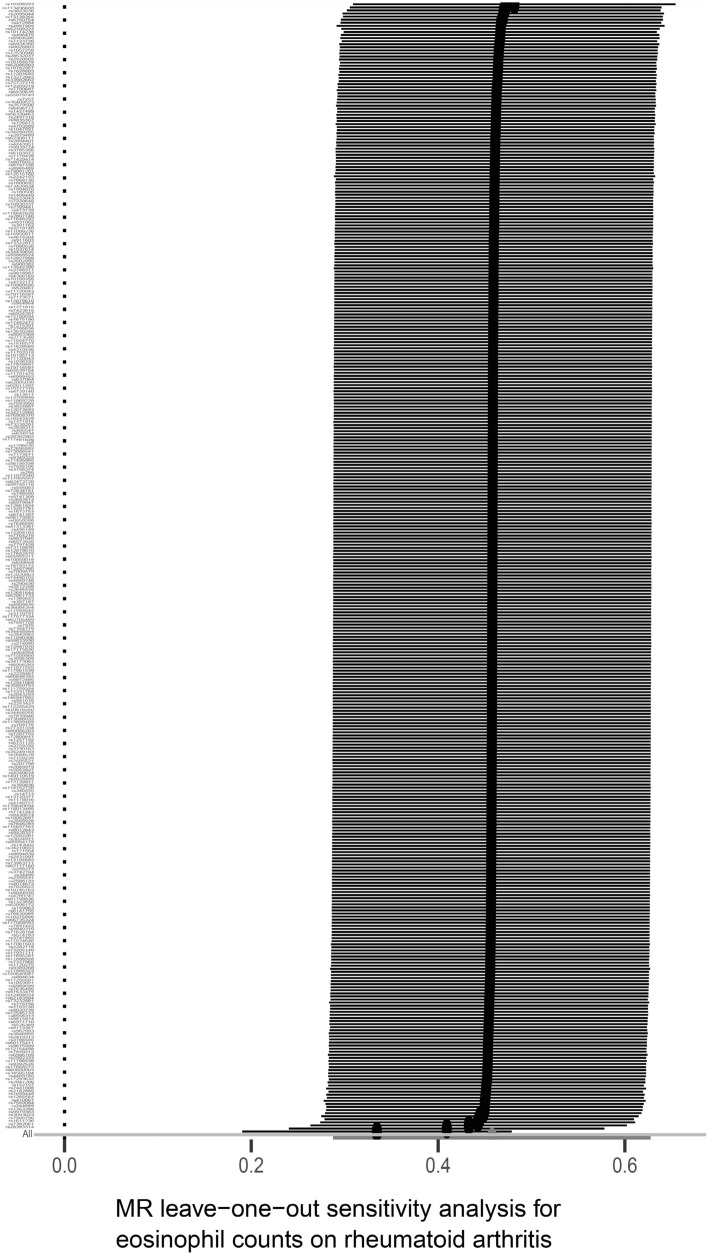
Sensitivity analysis of causal effect between eosinophil counts and RA based on the leave-one-out approach.

In this study, a variety of sensitivity analysis methods were used. First, Cochran's Q-test assessed heterogeneity between individual SNP estimates. For heterogeneity, it was observed in all outcomes: neutrophil counts (*p* = 0), lymphocyte counts (*p* = 9.69 × 10^–274^), monocyte counts (*p* = 9.50 × 10^–44^), eosinophil counts (*p* = 0) and basophil counts (*p* = 1.94 × 10^–51^), and white blood cell counts (*p* = 4.74 × 10^–280^). Therefore, we choose the random effects model to evaluate the causal effect^27^. Second, we used the MR-Egger intercept method to test the horizontal pleiotropy of IVs. There was no horizontal pleiotropy (*p* > 0.05) for each result. Then, Map funnels and forests show no presence of pleiotropy (Figs. S2, S3)^28^**.** At last, in the "leave-one-out" analysis, eosinophil counts were to the right of the 0 vertical lines (Fig. 4), indicating the classification result was deterministic.

## Discussion

In this study, we performed a univariate versus multivariate analysis to assess the causal relationship between types of blood leukocyte counts and RA. Univariate MR analysis revealed that eosinophils had a causal relationship with RA. The causal relationship between eosinophils and RA was more robust after correcting the interaction between different leukocyte traits^16^.

The increase in eosinophils predicts higher disease activity in RA, consistent with previous studies^29,30^. Eosinophils regulate adaptive immune responses and play an essential role in inflammatory and autoimmune diseases^31^. Observational studies have documented persistent increased eosinophilia in patients with RA^11,32^. The survey by Dewi Guellec et al.^8^ found that patients with mild eosinophilia were less responsive to treatment.The role of eosinophils on risk factors may be related to the eosinophil cationic protein (ECP), mediators stored in granules throughout the cytoplasm synthesized by eosinophils^33^. ECP, released from the activated eosinophils, is a ribonuclease with neurotoxic, cytotoxic, fibrosis-promoting, and immunomodulatory functions. Eosinophils release ECP in two ways: antibody-dependent (immunoglobulin) and antibody-independent (C3 and C5 complement components) activation of eosinophil degranulation^34,35^. Signaling by ECP induces the release of several pro-inflammatory factors/chemokines, including C30L and S100 proteins^36^, and may cause inflammation in a variety of tissue types^36^. The upregulation of CD30L is considered involved in RA pathogenesis^37^. We speculate that this is a possible mechanism of CD30L involvement in the immune process of RA. Moreover, previous studies have demonstrated that CD30 values are higher in active RA patients than in inactive RA and are directly related to rheumatoid factor serum titer^38,39^.

Due to the dual nature of eosinophils as pro-inflammatory and pro-resolving cells^40^, the association of eosinophils with the development of RA is still controversial. Darja Andreev et al.^12^ found that eosinophils’ pro-resolving properties in RA. Eosinophils promote the resolution of inflammation by producing pro-resolving lipid mediators through the 1/12-LOX-mediated biosynthetic pathway^41^. It was also demonstrated that eosinophils exert anti-inflammatory effects in arthritis by inducing M38 macrophage polarization by inhibiting the IκB/P2 MAPK signaling pathway^42^. Based on the different effects of eosinophils on RA, it can be speculated that eosinophils have subpopulations responsible for various biological functions. This conjecture has been confirmed that a population of regulatory eosinophils (rEos) exists in the joint that promotes the production of alternatively activated macrophages by generating IL-4, IL-13, and 12 / 15-lox-derived media^12^. Our MR analysis found that genetically predicted increased eosinophils were associated with an increased risk of RA, suggesting that the pro-inflammatory effect of eosinophils may be the primary driver of the increased risk of RA.

Moreover, our MR analysis did not indicate a causal relationship between other cell counts and RA except eosinophils. Although experimental studies have found that the absolute number of basophils increases in children with RA^43^, the causality remains unclear. Our univariate MR analysis confirms several observational studies, but after correction by multivariate MR analysis, this potential causality is no longer reliable. Previous studies may be limited by confounding factors and reverse causality. Studies have demonstrated an association between peripheral blood neutrophil-to-lymphocyte ratio (NLR), lymphocyte-to-monocyte ratio (LMR), and RA^44,45^, but our study did not find a causal relationship between neutrophil counts, lymphocyte counts, monocyte counts, and RA, indicating that NLR and LMR may be the main factor reflecting the potential pathogenesis of RA and disease progression. More studies are needed to confirm this.

Our study used the MR method to mitigate confounding bias and removed confounding factors by PhenoScanner. The robustness of the results was ensured by using three MR analysis techniques and a detailed sensitivity analysis. The large sample size from the white blood cell counts provided the strength of the tool variables (F statistic > 10). A limitation of this study is the need for detailed phenotypic data in available GWAS, which prevented us from testing the association of specific eosinophilic subtypes with RA, such as the risk of RA with rEOS counts as an exposure factor. In the future, more comprehensive GWAS data may allow stratified analyses of different cell subtypes.

## Conclusions

The current study remains controversial regarding the association of leukocytes with RA. Our MR study found that a higher genetically predicted risk of RA was associated with increased eosinophils but not with neutrophils, basophils, monocytes, and neutrophils. The association of RA with eosinophils may differ by different cell subsets, and more studies are needed to explore eosinophils' effects on the pathogenesis of RA.

### Supplementary Information

Supplementary Information 1.

Supplementary Information 2.

## Data Availability

The datasets used and/or analyzed during the current study are available from the corresponding author on reasonable request.
