# Supplementary material for: Genetic evidence for causal effects of leukocyte counts on risk for rheumatoid arthritis
Source: Sci Rep. 2023 Nov 26;13:20768. doi: 10.1038/s41598-023-46888-1 (PMC10679084; doi:10.1038/s41598-023-46888-1)
Supplement: Supplementary file 1 — Supplementary Information 1. [file 41598_2023_46888_MOESM1_ESM.docx]

Genetic evidence for causal effects of leukocyte counts on risk for rheumatoid arthritis

*Jin-Mei You^1#^, Yao-Chen Zhang^3,4#^, Ke-Yi Fan^3,4^, Shang-Kai Bai^3,4^, Zi-Yu Zhang^3,4^, He-Yi Zhang^3,4^, Ting Cheng^2,3,4^, Yue-Hong Huo^5^, Cai-Hong Wang^2,3,4^, Xiao-Feng Li^2,3,4^, Sheng-Xiao Zhang^2,3,4*^*

*^1^Department of clinicallaboratory, The Second Hospital of Shanxi Medical University, Taiyuan, Shanxi Province, China.*

*^2^Department of Rheumatology, The Second Hospital of Shanxi Medical University, Taiyuan, Shanxi Province, China.*

*^3^Shanxi Provincial Key Laboratory of Rheumatism Immune Microecology, Taiyuan, Shanxi Province, China.*

*^4^Key Laboratory of Cellular Physiology at Shanxi Medical University, Ministry of Education, Taiyuan, Shanxi Province, China.*

*^5^ Department of Rheumatology, The Fifth People’s Hospital of Datong, Datong, Shanxi Province, China.*

*^#^Jin-Mei You and Yao-Chen Zhang were co-senior authors and contributed equally to this work*

*^*^Correspondence To* *Sheng-Xiao Zhang, Department of Rheumatology, Second Hospital of Shanxi Medical University, Taiyuan 030001, E-mail:* [*zhangshengxiao1@sxmu.edu.cn,*](mailto:zhangshengxiao1@sxmu.edu.cn,) *Tel: 86-18734823329*

## Supplementary Figures


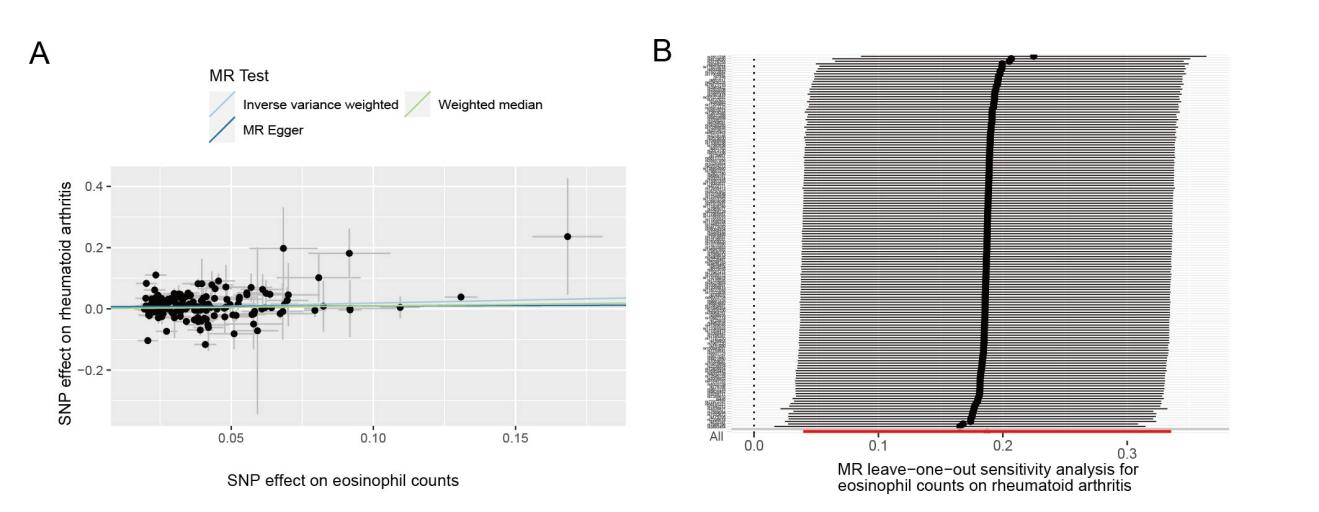


**Supplementary Figure 1.** Scatter plot and plot of leave-one-out in the validation group. A. The scatter plot of three mendelian randomization methods assessing the effect of eosinophil counts on RA. B. Sensitivity analysis of causal effect between eosinophil counts and RA based on the leave-one-out approach.


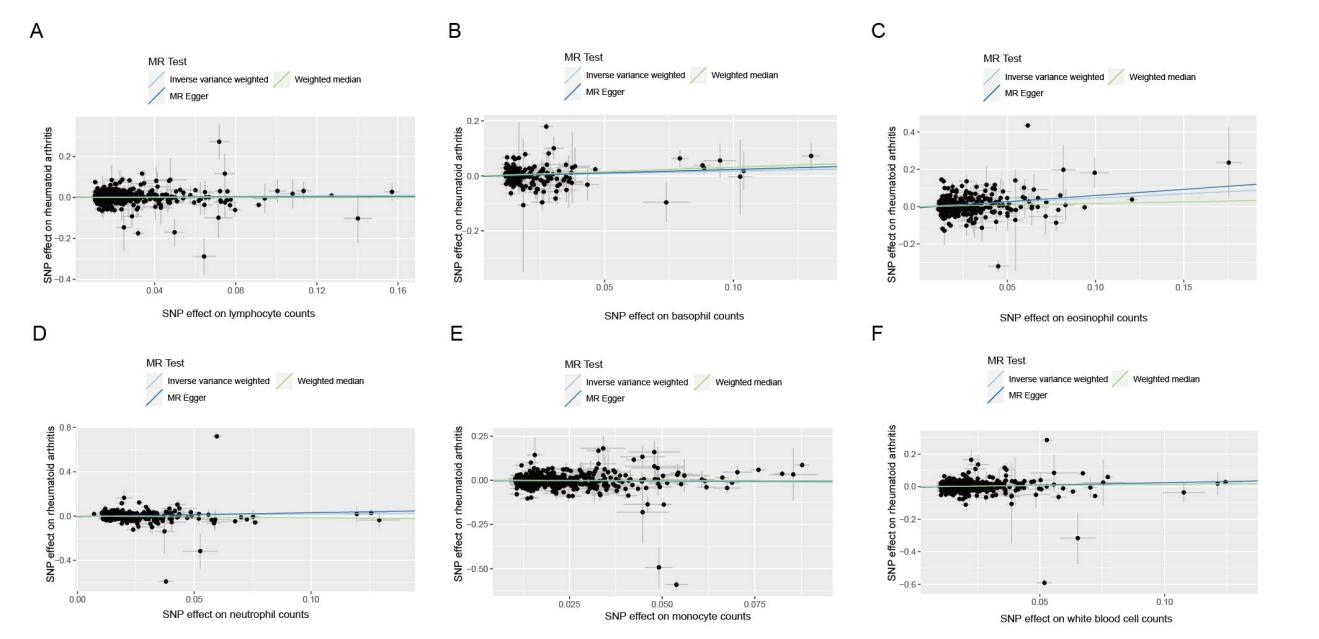


**Supplementary Figure 2.** The scatter plot of three mendelian randomization methods assessing the effect of types of blood leukocyte counts on RA. A. Lymphocyte counts; B. Basophil counts; C. Eosinophil counts; D. Neutrophil counts; E. Monocyte counts; F. White blood cell counts.


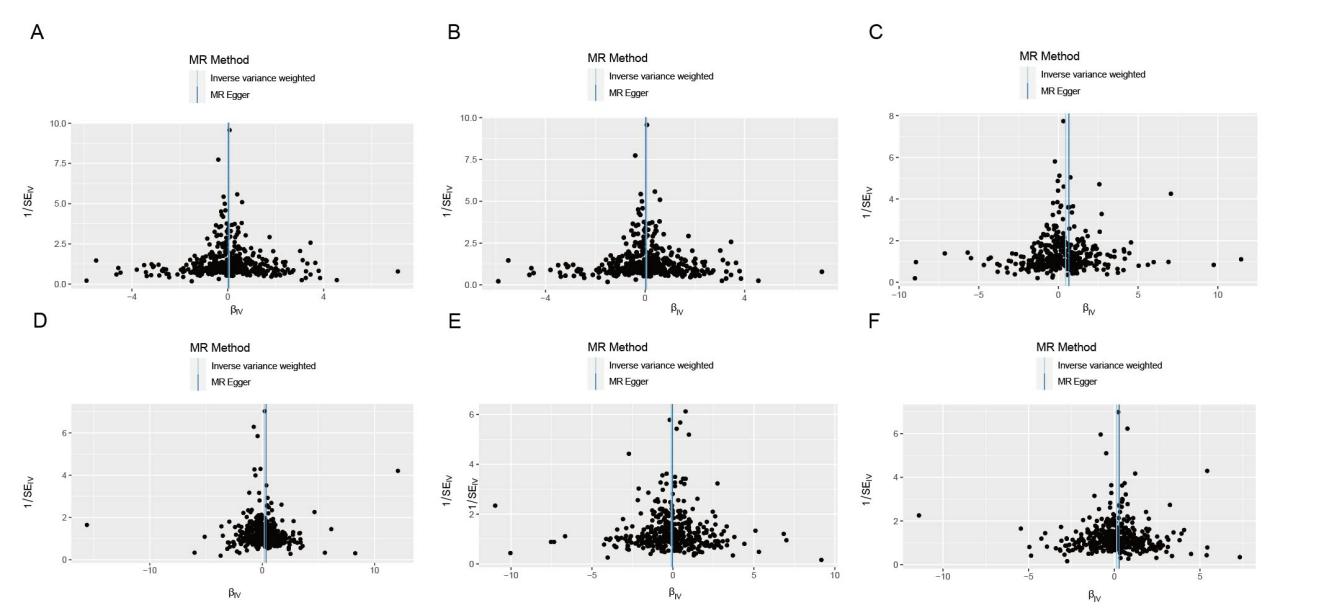


**Supplementary Figure 3.** The Funnel plots of mendelian randomization. A. Lymphocyte counts; B. Basophil counts; C. Eosinophil counts; D. Neutrophil counts; E. Monocyte counts; F. White blood cell counts.
